# Whole-brain gray matter maturation trajectories associated with autistic traits from adolescence to early adulthood

**DOI:** 10.1007/s00429-023-02710-2

**Published:** 2023-10-11

**Authors:** Guillaume Gros, Ruben Miranda Marcos, Anthony Latrille, Ana Saitovitch, Fanny Gollier-Briant, Philippe Fossati, Liane Schmidt, Tobias Banaschewski, Gareth J. Barker, Arun L. W. Bokde, Sylvane Desrivières, Antoine Grigis, Hugh Garavan, Penny Gowland, Andreas Heinz, Rüdiger Brühl, Jean-Luc Martinot, Marie-Laure Paillère Martinot, Eric Artiges, Frauke Nees, Dimitri Papadopoulos Orfanos, Luise Poustka, Sarah Hohmann, Nathalie Holz, Juliane H. Fröhner, Michael N. Smolka, Nilakshi Vaidya, Henrik Walter, Robert Whelan, Gunter Schumann, Hervé Lemaitre, Hélène Vulser, for the IMAGEN Consortium, Eric Artiges, Semiha Aydin, Christine Bach, Tobias Banaschewski, Alexis Barbot, Gareth Barker, Arun Bokde, Nadège Bordas, Zuleima Bricaud, Uli Bromberg, Ruediger Bruehl, Christian Büchel, Anna Cattrell, Patricia Conrod, Sylvane Desrivieres, Tahmine Fadai, Irina Filippi, Herta Flor, Vincent Frouin, André Galinowski, Jürgen Gallinat, Hugh Garavan, Fanny Gollier-Briant, Chantal Gourlan, Penny Gowland, Stella Guldner, Andreas Heinz, Bernd Ittermann, Tianye Jia, Hervé Lemaitre, Jean-Luc Martinot, Jessica Massicotte, Ruben Miranda, Kathrin Müller, Frauke Nees, Charlotte Nymberg, Marie Laure Paillère Martinot, Tomas Paus, Zdenka Pausova, Jean-Baptiste Poline, Luise Poustka, Jan Reuter, John Rogers, Barbara Ruggeri, Anna S Sarvasmaa, Christine Schmäl, Gunter Schumann, Maren Struve, Michael Smolka, Wolfgang Sommer, Hélène Vulser, Henrik Walter, Robert Whelan

**Affiliations:** 1 Control-Interoception-Attention Team, Hôpital Pitié-Salpêtrière Paris, Brain Institute, Inserm/CNRS/Sorbonne University, UMR 7225/U1127, Paris, France; 2 Department of Adult Psychiatry, Centre du Neurodéveloppement Adulte, AP-HP.Sorbonne Université, Pitié-Salpêtrière Hospital, 47-83 Boulevard de L’Hôpital, 75013 Paris, France; 3 Institut Des Maladies Neurodégénératives, UMR 5293, CNRS, CEA, Université de Bordeaux, 33076 Bordeaux, France; 4 Department of Pediatric Radiology, Necker-Enfants Malades Hospital, AP-HP, Université Paris Cité, Imagine Institute, INSERM U1299, UMR 1163, Paris, France; 5 Unité Diagnostique Autisme Ados-Jeunes Adultes (UD3A), CHU and Universite de Nantes, Fondation FondaMental, Nantes, Créteil France; 6 Department of Child and Adolescent Psychiatry and Psychotherapy, Medical Faculty Mannheim, Central Institute of Mental Health, Heidelberg University, Square J5, 68159 Mannheim, Germany; 7 Department of Neuroimaging, Institute of Psychiatry, Psychology and Neuroscience, King’s College London, London, UK; 8 Discipline of Psychiatry, School of Medicine and Trinity College Institute of Neuroscience, Trinity College Dublin, Dublin, Ireland; 9 Centre for Population Neuroscience and Precision Medicine (PONS), Institute of Psychiatry, Psychology and Neuroscience, SGDP Centre, King’s College London, London, UK; 10 NeuroSpin, CEA, Université Paris-Saclay, 91191 Gif-Sur-Yvette, France; 11 Departments of Psychiatry and Psychology, University of Vermont, Burlington, VT 05405 USA; 12 Sir Peter Mansfield Imaging Centre School of Physics and Astronomy, University of Nottingham, University Park, Nottingham, UK; 13 Department of Psychiatry and Psychotherapy CCM, Charité–Universitätsmedizin Berlin, corporate member of Freie Universität Berlin, Humboldt-Universität Zu Berlin, and Berlin Institute of Health, Berlin, Germany; 14 Physikalisch-Technische Bundesanstalt (PTB), Braunschweig, Berlin, Germany; 15 Institut National de La Santé Et de La Recherche Médicale, INSERM U 1299 “Trajectoires Développementales and Psychiatrie”, University Paris-Saclay, CNRS, Ecole Normale Supérieure Paris-Saclay, Centre Borelli, Gif-Sur-Yvette, France; 16 Department of Child and Adolescent Psychiatry, AP-HP. Sorbonne University, Pitié-Salpêtrière Hospital, Paris, France; 17 Psychiatry Department, EPS Barthélémy Durand, Etampes, France; 18 Institute of Cognitive and Clinical Neuroscience, Medical Faculty Mannheim, Central Institute of Mental Health, Heidelberg University, Square J5, Mannheim, Germany; 19 Institute of Medical Psychology and Medical Sociology, University Medical Center Schleswig Holstein, Kiel University, Kiel, Germany; 20 Department of Child and Adolescent Psychiatry, Center for Psychosocial Medicine, University Hospital Heidelberg, Heidelberg, Germany; 21 Department of Child and Adolescent Psychiatry, Psychotherapy and Psychosomatics, University Medical Center Hamburg-Eppendorf, Hamburg, Germany; 22 Department of Psychiatry and Neuroimaging Center, Technische Universität Dresden, Dresden, Germany; 23 Centre for Population Neuroscience and Stratified Medicine (PONS), Department of Psychiatry and Neuroscience, Charité Universitätsmedizin Berlin, Berlin, Germany; 24 School of Psychology and Global Brain Health Institute, Trinity College Dublin, Dublin, Ireland; 25 Centre for Population Neuroscience and Precision Medicine (PONS), Institute for Science and Technology of Brain-Inspired Intelligence (ISTBI), Fudan University, Shanghai, China

**Keywords:** Adolescent, Autistic traits, MRI, Gray matter, Longitudinal, Women

## Abstract

A growing number of evidence supports a continued distribution of autistic traits in the general population. However, brain maturation trajectories of autistic traits as well as the influence of sex on these trajectories remain largely unknown. We investigated the association of autistic traits in the general population, with longitudinal gray matter (GM) maturation trajectories during the critical period of adolescence. We assessed 709 community-based adolescents (54.7% women) at age 14 and 22. After testing the effect of sex, we used whole-brain voxel-based morphometry to measure longitudinal GM volumes changes associated with autistic traits measured by the Social Responsiveness Scale (SRS) total and sub-scores. In women, we observed that the SRS was associated with slower GM volume decrease globally and in the left parahippocampus and middle temporal gyrus. The social communication sub-score correlated with slower GM volume decrease in the left parahippocampal, superior temporal gyrus, and pallidum; and the social cognition sub-score correlated with slower GM volume decrease in the left middle temporal gyrus, the right ventromedial prefrontal and orbitofrontal cortex. No longitudinal association was found in men. Autistic traits in young women were found to be associated with specific brain trajectories in regions of the social brain and the reward circuit known to be involved in Autism Spectrum Disorder. These findings support both the hypothesis of an earlier GM maturation associated with autistic traits in adolescence and of protective mechanisms in women. They advocate for further studies on brain trajectories associated with autistic traits in women.

## Introduction

Although international classifications still distinguish Autism Spectrum Disorder (ASD) on a categorical basis, a growing number of evidence supports a continued distribution of autistic traits in the general population (Whitehouse et al. 2011). Autistic traits have been reported to be associated with greater psychosocial difficulties and poorer mental health (Lai et al. 2014) and seem to share genetic vulnerability factor with ASD, with high prevalence of these traits in family members of people with ASD (Ronald and Hoekstra 2011; Robinson et al. 2011a, b; Bralten et al. 2018). However, cerebral bases of autistic traits remain largely unknown.

In people with ASD, numerous evidence has shown structural volumetric abnormalities in the frontal and temporal cortex, notably in the superior temporal sulcus, the fusiform gyrus and the insula (Boddaertet al. 2004; McAlonan et al. 2005; Hadjikhani et al. 2006; Brun et al. 2009; Greimel et al. 2013; Ecker et al. 2012; Ecker et al. 2013; Ecker et al. 2015). A relatively recent mega-analysis from the ENIGMA ASD Working Group reported that participants with ASD had significant smaller volumes of the putamen, the pallidum, the amygdala and the nucleus accumbens. In addition, they reported higher cortical thickness in the middle and superior frontal, orbitofrontal, inferior frontal, and posterior cingulate areas and lower cortical thickness in the temporal, entorhinal, and parahippocampal areas (van Rooij et al. 2018). Another recent meta-analysis revealed thicker cortical thickness in the right superior temporal, left middle temporal, anterior cingulate/paracingulate gyri, and right superior frontal (medial orbital part) gyri and higher cortical thinning the left parahippocampal gyrus, the right precentral gyrus and the left middle frontal gyrus (Shen et al. 2022).

Taken together, although controversial (Schumann et al. 2010a, b; Aylward et al. 1999; Groen et al. 2010; Hardan et al. 2001; Herbert et al. 2003; Bellani et al. 2013; Barnea-Goraly et al. 2014; Brambilla et al. 2003; Nickl-Jockschat et al. 2012; Rojas et al. 2006), these results suggest abnormalities in regions of the social brain (orbitofrontal cortex, temporal cortex, amygdala, fusiform gyrus) and the reward circuit (fronto-striatal). These characteristics may differ between sexes (Bloss and Courchesnes 2007; Craig et al. 2007; Schaer et al. 2015; Retico et al. 2016; Ecker et al. 2017; Walsh et al. 2021) with generally lower cortical thickness or volume associated with ASD in females and greater cortical thickness or volume in males (Walsh et al. 2021). However, because of the sex ratio in favor of men (Loomes et al. 2017), very few studies have included women or were specific to women.

Some other findings suggest that ASD is not associated with fixed brain abnormalities but rather support the hypothesis of a complex dynamic disorder with abnormal neuro-developmental trajectories of the brain. Several studies reported a whole brain volume increase at a faster rate during childhood, such that volume peaked in early adolescence followed by a higher decrease during early adolescence to meet the typical development curve between 10 and 15 years of age (Lange et al. 2015; Sussman et al. 2015; Mak-Fan et al. 2013; Stigler et al. 2011; Courchesne et al. 2001). Regarding cortical thickness, ASD seems to be also associated with an abnormal development with a more important thickening during early childhood, followed by an earlier thinning in later childhood leading to a decelerated thinning in early adulthood (Sussman et al. 2015; Doyle-Thomas et al. 2013; Raznahan et al. 2010; Zielinski et al. 2014), yet with important region specificities (Sussman et al. 2015; Doyle-Thomas et al. 2013; Raznahan et al. 2010; Zielinski et al. 2014). Again, distinct neurodevelopmental patterns seem to differentiate females from males with ASD (Walsh et al. 2021).

Regarding autistic traits, few studies have suggested a dimensional continuity in brain specificities along the spectrum. Using the Social Responsiveness Scale (SRS) (Constantino and Gruber 2005), in 6–10-year-old children, Blanken et al. (2015) observed a decreased gyrification in a large cluster involving the left hemisphere temporal and precuneus regions associated with autistic traits. Using the same questionnaire an association between autistic traits and cortical thickness was also found in the right superior temporal gyrus and the insula in 26 typically developing male adolescents (Tu et al. 2016). Regarding gray matter (GM) volume, results showed that autistic traits were associated with smaller volumes in the left posterior cingulate in young adults (Geurts et al. 2013). Using the Autism-Spectrum Quotient, a voxel-based morphometry (VBM) study revealed smaller GM volume in the insula associated with the 'prosocial’ dimension in males only (Saito et al. 2014). Another VBM study revealed higher GM volume in the orbitofrontal cortex, the cuneus, the hippocampus and parahippocampus and smaller GM volume in the inferior temporal cortex, the insula, and the amygdala in young men and women with high autistic traits measured by the Empathizing Quotient and the Systemizing Quotient (Focquaert and Vanneste 2015). A recent study from the IMAGEN consortium used a region of interest design to show an association between SRS scores and volumetric changes in the amygdala, the cerebellum, and the hippocampus in male and female adolescents, and in the putamen specifically in boys (Nees et al. 2022). Interestingly, brain correlates of autistic traits might also differ between sex, as suggested by a recent twin study conducted in 74 same-sex twin pairs (31 female pairs, 49 monozygotic) (Cauvet et al. 2019). Within female twin pairs increases in autistic traits (measured by the SRS) were associated with smaller cortical volume and surface area in numerous temporal and frontal regions linked to social communication. In contrast, autistic traits related less extensively with cortical volume and surface area in male twin pairs. associated with autistic traits in boys were much less extensive.

Finally, only one study investigated longitudinal brain changes associated with autistic traits. In 56 males (age 3–39 years at their first scan) followed-up for 7 years, a significant SRS score by age interaction was found in the left temporal pole and the left superior parietal cortex (Prigge et al. 2018).

Altogether, these results suggest specific brain trajectories associated with autistic traits, particularly during adolescence. However, only one study investigated longitudinal aspects, and none used a whole brain volumetric design. Moreover, sex effects of brain trajectories associated with autistic traits remain totally unexplored.

In the present neuroimaging study, we, therefore, used whole brain voxel-based morphometry to investigate whether autistic traits in the general population, measured by the SRS, are associated with longitudinal GM trajectories during adolescence (between 14 and 22 years). Due to major differences between boys and girls in terms of both prevalence of ASD and brain maturation in this critical period of adolescence (Shaw et al. 2008) the influence of sex on the age-related GM trajectories was also tested.

In addition, we aimed to have a comprehensive understanding of the distinct dimensions of autistic traits associated with GM trajectories.

## Methods

### Participants

Neuroimaging and clinical data were obtained from an existing large European multi-site longitudinal study of adolescent development (https://imagen-project.org) across eight European sites in France, United Kingdom, Ireland, and Germany, which includes 2,223 adolescents recruited in schools around age 14 (standard deviation = 0.41; age range: 12.9–15.7). A detailed description of recruitment and assessment procedures, with exclusion and inclusion criteria, has been published elsewhere (Schumann et al. 2010a, b). Notably, any obvious psychopathology at baseline (e.g., bipolar disorder, schizophrenia, or major neuro-developmental disorders), any severe medical somatic conditions, any history of head injuries, and any contraindications for magnetic resonance imaging (MRI) constituted exclusion criteria.

The study was approved by the ethics committees in all participating institutions. Written informed assent and consent were obtained, respectively, from all adolescents and their parents after complete description of the study.

Participants were followed up 2 years later for clinical assessment and 8 years later for clinical and neuroimaging assessment.

**Fig. 1:**
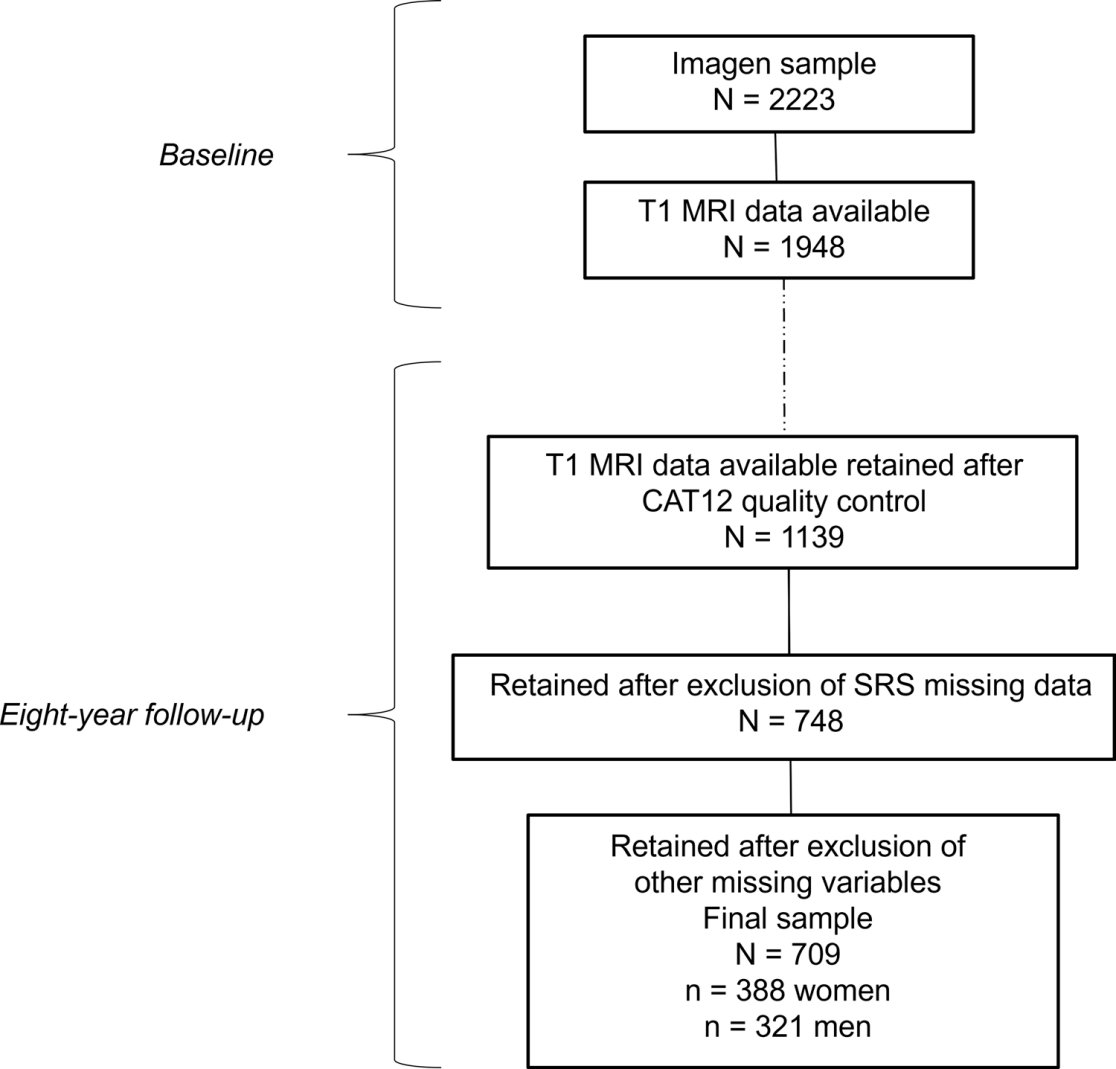
Flow chart of the study population

A total of 1182 had available MRI data at both age 14 and 22. We excluded participants who did not pass the MRI quality control, those who did not complete the SRS questionnaire and those with missing data (Fig. 1). In this study, we present data from the remaining 709 participants (*n* = 388 women).

### MRI data

All participants underwent MRI examination on 3 Tesla scanners (General Electric, Siemens, and Philips) at 14 and 22 from the 8 European sites. High-resolution structural T1-weighted images were obtained using a standardized 3D T1-weighted magnetization prepared rapid acquisition echo (MPRAGE) sequence based on the ADNI protocol (http://adni.loni.usc.edu/methods/mri-analysis/mri-acquisition). All images passed a visual quality-control carried out by two independent raters to identify and discard images with major artifacts, such as motion, aliasing, ghosting, spikes, low signal to noise ratio. VBM was performed with the Computational Anatomy Toolbox (CAT, http://www.neuro.uni-jena.de/cat/) of Statistical Parametric Mapping 12 software (SPM12 www.fil.ion.ucl.ac.uk/spm) (Ashburner and Friston 2000).

T1-weighted images were preprocessed using a dedicated longitudinal pipeline to properly probe longitudinal (between age 14 and age 22) and cross-sectional (at both age 14 and 22) structural variations with CAT12. CAT12 provides a groupwise intra-subject modeling framework for longitudinal structural MRI data, which combines diffeomorphic and rigid-body registrations, tissue segmentation, and a correction of intensity inhomogeneity (Ashburner and Ridgway 2013). All images passed a post-preprocessing standardized CAT12 quality control. Images were retained if their quotation was D- (“sufficient”) or better corresponding to a weighted average image quality rating (IQR) > 60 (https://neuro-jena.github.io/cat12-help/).

The normalized, segmented and modulated GM images were smoothed using a 10-mm full-width at half-maximum (FWHM) Gaussian kernel. Global GM, white matter (WM), and cerebro-spinal fluid (CSF) volumes were computed for each participant at both age 14 and 22. Total Intracranial Volume (TIV) was defined as the sum of GM, WM, and CSF volumes.

### Baseline characteristics

#### Autistic traits

Autistic traits were assessed at age 16 with the Social Responsiveness Scale (SRS), in its parent-reported form. The SRS is a well-validated questionnaire in children and adolescents aged 4 to 18 referring to five autistic dimensions: social communication, social motivation, social awareness, social cognition, restricted or repetitive behavior and interests (Constantino and Gruber 2005). Psytools software (Delosis Ltd, London, UK) was used to conduct the SRS assessment via its internet-based platform. Raw scores of SRS score and sub-scores are presented in Table 1 and were used in the analyses.

**Table 1:** Clinical characteristics and global brain volumes of the 709 participants

	Baseline						Follow-up					
	Women (*n* = 388)		Men (*n* = 321)				Women (*n* = 388)		Men (*n* = 321)			
	*m*^a^	sd^b^	*m*	sd	*t*	*p*	m	sd	*m*	sd	*t*	*p*
Age	14.38	0.45	14.36	0.39	0.67	0.50	22.40	0.56	22.39	0.61	0.29	0.78
SRS^c^ scores												
SRS total	24.07	15.46	25.77	17.28	– 1.37	0.17						
Social communication	7.61	5.92	8.79	6.98	– 2.39	0.02						
Social cognition	4.13	3.64	4.3	3.61	– 0.62	0.54						
Social awareness	4.58	2.56	5.04	2.67	– 2.37	0.02						
Social motivation	5.23	3.56	5.26	3.90	– 0.09	0.93						
RRBI^d^	2.53	2.93	2.39	3.01	– 0.61	0.54						
Total IQ^e^	109.97	11.45	111.49	12.18	– 1.71	0.09						
IQR^f^	81.69	3.63	81.23	4.28	1.52	0.13						

	*n*	%	*n*	%	*χ*^*2*^	*p*
Center					4.01	0.78
Berlin	53	13.66	35	10.90		
Dresden	44	11.34	40	12.46		
Dublin	34	8.76	28	8.72		
Hamburg	50	12.86	52	16.2		
London	50	12.86	48	14.95		
Mannheim	54	13.92	39	12.15		
Nottingham	54	13.92	44	13.71		
Paris	49	12.63	35	10.90		

Brain tissue (in cm^3^)	*m*	sd	*m*	sd	*t*	*p*	*m*	sd	*m*	sd	*t*	*p*
Gray matter	699.52	52.34	788.81	59.33	21.03	< 0.001	640.68	46.16	711.06	52.83	– 18.69	< 0.001
White matter	471.08	44.64	536.77	50.13	18.24	< 0.001	476.86	46.80	550.38	52.19	– 19.56	< 0.001
Cerebro-spinal fluid	228.28	40.19	240.80	45.17	3.86	< 0.001	225.57	42.19	276.14	49.35	– 14.49	< 0.001
Total intracranial volume	1399.39	103.95	1567.15	1145.88	20.10	< 0.001	1343.56	99.32	1538.33	116.89	– 23.62	< 0.001

^a^*m* mean

^b^*sd* standard deviation

^c^*SRS* social responsiveness scale

^d^*RRBI* restricted or repetitive behavior and interests

^e^*IQ* intelligence quotient

^f^*IQR* weighted average image quality rating

#### Screening for intelligence

Total Intelligence Quotient (IQ) was estimated at age 14 using the Wechsler Intelligence Scale for Children (WISC IV, Wechsler 2003).

### Statistical analyses

#### Principal analysis

We first explored a potential sex effect in the associations between total SRS score and global GM volume variations between ages 14 and 22 within the framework of a mixed design model using R software and lme4 package. Age, SRS and sex (as assigned at birth) as main factors, and age-by-SRS-by-sex interaction were incorporated as fixed effects in the model (i.e., the following interactions: age*SRS, age*sex, SRS*sex were covered by the three-way interaction). Subject intercept and center were included as random effect. IQ and TIV were used as covariates.

If the interaction term was statistically significant, the association between total SRS score and global GM age-related changes were tested in men and women separately using a mixed design model using the same covariates.

If the age-by-SRS-by-sex triple interaction term was statistically significant, the association between total SRS score and voxelwise age-related GM changes were tested in men and women separately.

Longitudinal (between ages 14 and 22) whole brain voxelwise analyses were carried out within CAT12. To test the longitudinal effect within CAT12, we used a flexible factorial design (i.e., mixed design) with time considered as within-subject and other factors as between-subject. Age, center, IQ, TIV, and IQR were entered as confounding variables. At the voxel-level, statistical significance was set to *p* < 0.05 Family Wise Error (FWE) corrected for multiple comparisons. Brain locations were reported as x, y, and z coordinates in the Montreal Neurologic Institute (MNI) space.

#### Secondary analyses

If the previous association between total SRS score and GM volume variations was significant in the previous voxelwise analysis, we investigated the association between each SRS dimension and voxelwise GM volume variations using the same methodology.

To further complete and add a comprehensive understanding of our results, cross-sectional whole brain voxelwise analyses (at both age 14 and 22) were carried out when a significant association between SRS (total or sub-score) and voxelwise GM volume variations was observed in previous principal and secondary analyses. We used the general linear model framework provided by SPM to test multiple regression models with age, center, IQ, TIV and IQR as covariates.

## Results

Clinical characteristics and global brain volumes of the 709 participants are described in Table 1.

IQR was not associated with total SRS score or with any SRS dimension.

### Principal analysis

#### Global GM variations

**Fig. 2:**
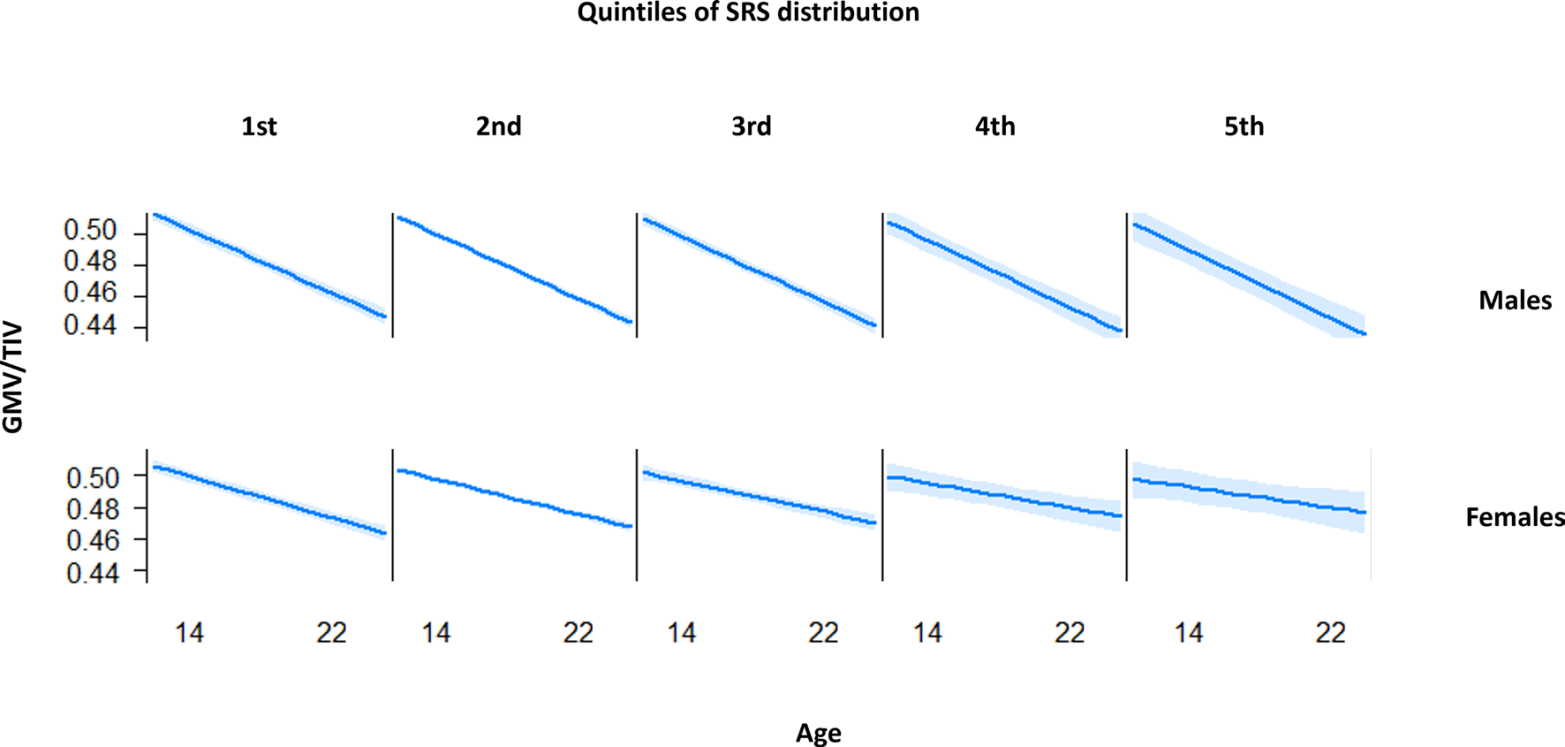
Plots representing the interaction age x Social Responsiveness Scale (SRS) total score x sex using R “effects” package. *GMV/TIV* gray matter volume/total intracranial volume, *SRS* social responsiveness scale total score

A significant age-by-SRS-by-sex triple interaction term was found for global GM volume variations between ages 14 and 22 (*p* = 0.01). GM volume variation correlated with SRS total score in women (p value of the interaction age x SRS = 0.02, *χ*^2^ = 5.20) but not in men (*p* = 0.63 *χ*^2^ = 0.23) (*p* for age x sex interaction < 0.001) (Fig. 2). For example, in women with the lowest scores of SRS (first quintile), we observed a decrease in global GM volume of – 5.26 cm^3^/year, while women with the highest scores of SRS (5th quintile) demonstrated a smaller decrease of GM volume: – 3.55 cm^3^/year. In contrast, males demonstrated higher GM decrease that was similar in the first (– 8.42 cm^3^/year) and the 5th quintile (– 8.67 cm^3^/year). Age x sex interaction was significant in each quintile of SRS (from the first to the 5th quintile: *χ*^2^ = 26.80, *p* < 0.001; *χ*^2^ = 16.72, *p* < 0.001; *χ*^2^ = 36.31, *p* < 0.001; *χ*^2^ = 24.13, *p* < 0.001; *χ*^2^ = 64.34, *p* < 0.001).

#### Voxelwise gray matter variations

**Fig. 3:**
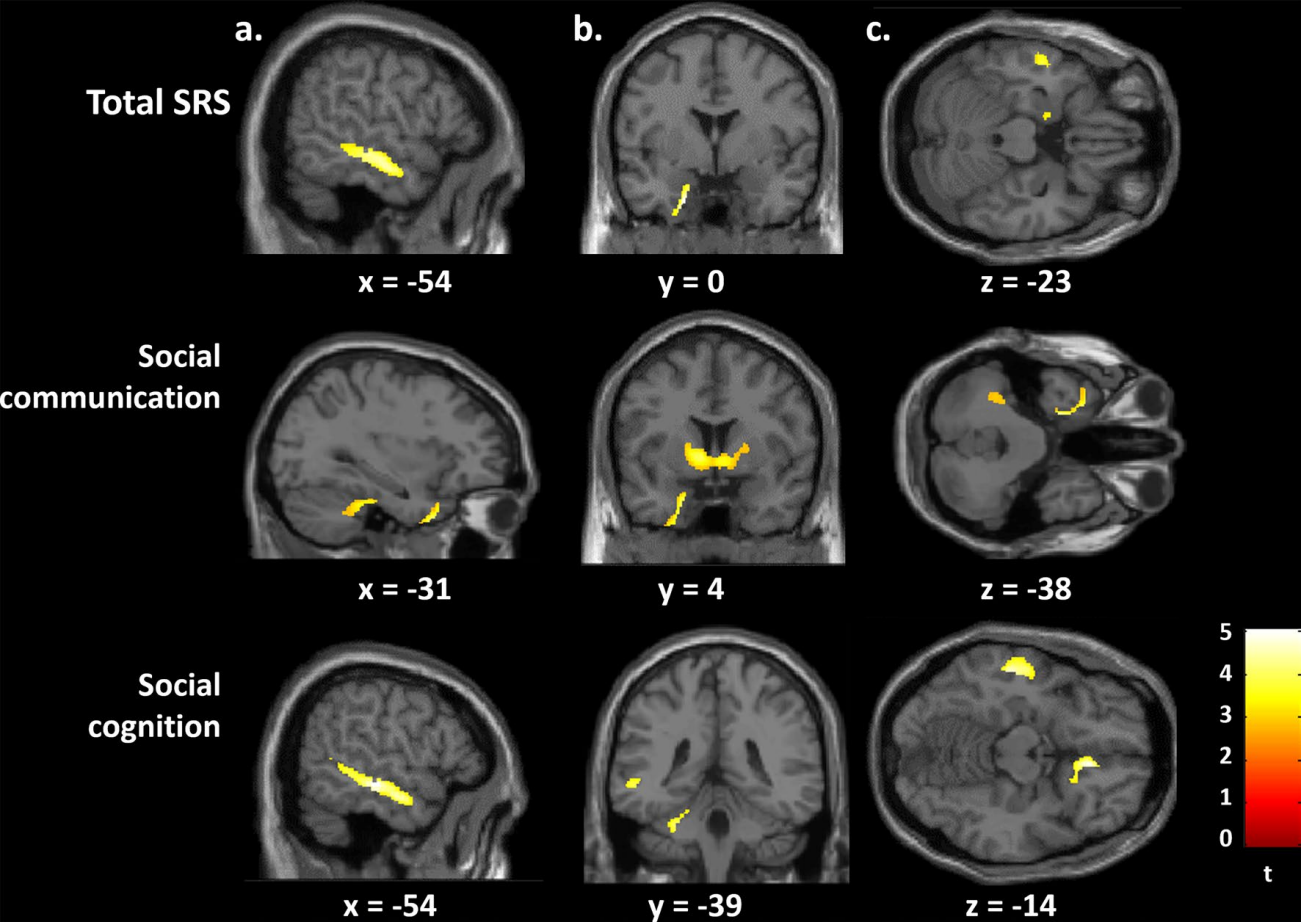
Brain regions where the Social Responsiveness Scale (SRS) total score, and the social communication and social cognition SRS sub-scores correlated with slower gray matter decrease between 14 and 22 in the 388 women. Images are presented with height threshold *p* < 0.001. Results are superimposed on a T1-weighted MRI scan. **a** Sagittal slice; **b** coronal slice; **c** transversal slice; *R* right. Localisation of peak-level at *p* < 0.05, FWE corrected in presented clusters: total SRS. **a** Left middle temporal gyrus, **b** left parahippocampus, **c** left parahippocampus and left middle temporal gyrus; social communication sub-score: **a** left superior temporal gyrus and left cerebellum, **b** left parahippocampus and left pallidum, **c** left parahippocampus, left superior temporal gyrus and left cerebellum; social cognition sub-score: **a** left middle temporal gyrus, **b** left middle temporal gyrus and left cerebellum, **c** left middle temporal gyrus and right ventromedial prefrontal/orbitofrontal cortex

In women, the whole brain longitudinal analysis revealed that higher SRS total score correlated with slower GM volume decrease between 14 and 22 in the left parahippocampal gyrus [Brodmann area (BA) 28/34/36] and in the left middle temporal gyrus (BA 21) (Table 2, Fig. 3).

**Table 2:** Longitudinal significant whole brain voxelwise correlation between gray matter volumes variations and SRS scores in women and significant corresponding cross-sectional correlations

Brain region	BA^a^	Cluster-level	k_FWEcor_^b^	k_uncor_^c^	MNI^d^ coordinates			Peak-level	*t*
		p_FWEcor_							
					*x*	*y*	*z*	p_FWEcor_	
Total SRS^e^									
Longitudinal (slower decrease)									
Left parahippocampus	28/34/36	0.023	31	533	– 20	0	– 34	0.002	5.11
Left middle temporal gyrus	21	0.018	48	738	– 56	– 14	– 16	0.014	4.68
Social communication sub-score									
Longitudinal (slower decrease)									
Left parahippocampus	28/34/36	0.013	74	618	– 20	– 2	– 34	0.014	5.79
Left superior temporal gyrus	38	0.029	19	(618)	– 30	12	– 42	0.017	4.64
Left pallidum		0.025	26	1147	– 9	4	0	0.019	4.60
Left cerebellum		0.039	6	427	– 27	– 34	– 30	0.028	4.51
Left middle temporal gyrus	21	0.046	1	473	– 56	– 14	– 16	0.050	4.36
Cross-sectional at age 14 (negative)									
Left parahippocampus	28/34/36	0.016	57	422	– 18	– 4	– 33	< 0.001	5.09
_			_		– 18	3	– 28	0.002	4.68
Social cognition sub-score									
Longitudinal (slower decrease)									
Left middle temporal gyrus	21	0.017	52	976	– 54	– 24	– 10	0.003	5.02
Right vmPFC^e^/OFC^f^	11/15	0.024	30	269	8	27	– 14	0.007	4.84
Left middle temporal gyrus	21	0.022	33	(976)	– 52	– 8	– 20	0.023	4.56
Left cerebellum		0.040	5	141	0	– 75	– 44	0.033	4.47
Left cerebellum		0.043	3	377	– 27	– 34	– 30	0.033	4.47
Left superior temporal gyrus	22	0.044	2	(976)	– 48	– 54	8	0.046	4.38

Height threshold *p* < 0.05 family wise error (FWE) corrected

^a^*BA* Brodmann area

^b^*k *_***FWE cor***_ cluster size at height threshold *p* < 0.05 Family Wise Error (FWE) corrected

^c^cluster size at height threshold *p* < 0.001 uncorrected, k numbers in parentheses indicate a previously mentioned cluster

^d^*MNI* Montreal neurologic institute coordinates in millimeters

^e^social responsiveness scale

^f^*vmPFC* ventromedial prefrontal cortex

^g^*OFC* orbitofrontal cortex. Longitudinal (slower decrease), SRS score is associated with slower decrease in this region between 14 and 22; cross-sectional (negative): SRS score is associated with smaller gray matter volume in this region

SRS total score did not correlate with any region of GM volume variation in men.

### Secondary analyses

#### SRS dimensions

**Fig. 4:**
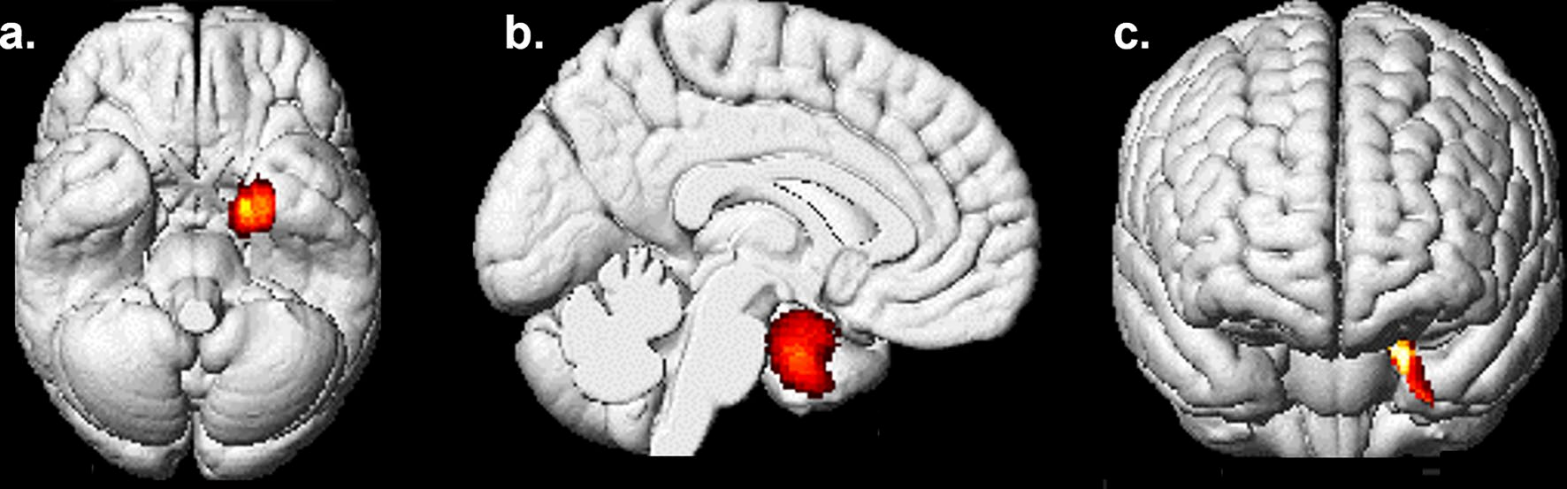
Inferior (**a**), sagittal (**b**) and anterior (**c**) view of the left parahippocampal cluster, where a negative volumetric correlation was found with the social communication SRS sub-score at age 14 in the 388 women

Longitudinal whole brain analyses revealed an association between higher SRS social communication sub-scores and slower GM volume decrease in the left parahippocampal gyrus (BA 28/34/36, Fig. 4), in the left superior temporal gyrus (BA 38), the left pallidum, the left cerebellum and the left middle temporal gyrus (BA 21) (Fig. 3, Table 2) in women. In addition, SRS social cognition sub-scores correlated with slower GM volume decrease in the left middle temporal gyrus (BA 21), the right ventromedial prefrontal (vmPFC) and orbitofrontal cortex (OFC) (BA 11/25), the left cerebellum and the left superior temporal gyrus (BA 22) in women (Fig. 3, Table 2). Other SRS sub-scores (awareness, motivation and restricted or repetitive behavior and interests) did not correlate with any region of GM volume variation in women.

#### Cross-sectional investigations

At age 14, SRS social communication sub-score correlated with lower GM volume in the same region in women. No significant association was observed regarding total or SRS social cognition sub-score.

At age 22, no significant association was observed, neither for SRS total score nor for any of the two sub-scores.

## Discussion

In the present study, we investigated for the first time the association between autistic traits in the general population and whole brain longitudinal GM volume changes during adolescence.

In women, we observed that higher autistic traits measured by the SRS were associated with slower GM volume decrease between age 14 and 22 globally and in the left parahippocampus and the left middle temporal gyrus. Left parahippocampal longitudinal variations appear to be related to social communication traits, whereas left middle temporal variations were more related to social cognition traits. In addition, social communication traits were associated with slower GM volume decrease between ages 14 and 22 in the left superior temporal gyrus and the left pallidum, and social cognition traits were associated with slower GM volume decrease in the right prefrontal ventromedial/orbitofrontal cortex. Both social communication and social cognition were associated with slower GM volume decrease in small clusters of the left cerebellum. Finally, we observed an association between social communication sub-score and smaller GM volume at age 14 in the left parahippocampus that was no longer present at age 22. No longitudinal association was found in men.

The present findings extend the very limited literature existing on brain structure development associated with autistic traits. Our results seem in line with previous findings on brain trajectories in ASD, namely, an accelerated brain maturation in childhood leading to an earlier peak-volume followed by a decelerated brain maturation in early adulthood to meet the typical development curve (Sussman et al. 2015; Doyle-Thomas et al. 2013; Raznahan et al. 2010; Zielinski et al. 2014). Indeed, we observed slower GM volume decrease between mid-adolescence and early adulthood associated with higher SRS scores. In addition, smaller GM volume in the left parahippocampus were associated with SRS social communication score at age 14 but not age 22. This could suggest that autistic traits are associated with an earlier brain maturation in regions involved in social reciprocity, and notably social communication, such as the parahippocampus. Unfortunately, we did not observe similar significant findings, of an association observed at age 14 but not 22, regarding other clusters of this study. However, on a more exploratory threshold (*p* < 0.001, uncorrected), we observed an association between SRS social communication sub-score and both smaller volumes in the left superior temporal gyrus and the right pallidum, that did not persist at age 22. At this more permissive threshold, smaller GM volume in several other regions were also associated with SRS scores at age 14 but not 22 (bilateral parahippocampus, left insula, left putamen for social cognition, left insula, left thalamus, bilateral parahippocampus, putamen, caudate head, bilateral fusiform, left putamen, bilateral caudate head for SRS total and social communication). Altogether, the results of the present study support the hypothesis of an earlier GM maturation associated with autistic traits in adolescence that normalizes in early adulthood. However, these results might also be seen as the result of a delay in brain development or indicative of prolonged development. Some discrepancies with the only previous report on longitudinal changes associated with autistic traits must be highlighted (Prigge et al. 2018). In the study of Prigge et al., 91 participants with ASD and 56 controls aged 3–39 years at first scan were scanned up to three times over a 7-year period to examine cortical thickness in relation to SRS score. The authors reported that SRS scores had a greater degree of age-related cortical thinning in the left temporal pole—where we found slower increase. However, the population was different from that of our study: (1) the age range was larger than in our study; (2) they included participants with ASD; (3) they studied cortical thickness and not volumes and (4) as discussed in the following paragraph, they included only males. In addition, the authors reported reduced age-related changes in the left superior parietal cortex.

Another important finding of the present study is the absence of results in male participants. Although males are predominantly represented in MRI studies on ASD, some data suggested sex-by-diagnosis effects (Craig et al. 2007; Sussman et al. 2015). Interestingly, such an effect was reported in the bilateral parahippocampal (BA 28/34), the middle temporal lobe (BA 21/22) and the vmPFC/OFC (Schaer et al. 2015; Ecker et al. 2019), that are regions in which we observed slower GM decrease in females—but not in males—associated with higher autistic traits. A recent meta-analysis (Walsh et al. 2021) confirmed and extended this sex-by-diagnosis effect within regions associated with the limbic, default mode, visual, and auditory/language regions networks. This effect was mainly explained by atypically lower volume or thickness in females with ASD compared to trends toward higher values in males with ASD, with an age-dependency of regional sex-by-diagnosis effects suggested by the authors. More generally, a growing literature supports the existence of greater extend of structural brain alterations associated with autism and autistic traits in females (Bloss and Courchesnes 2007; Schumann et al. 2009; Schumann et al. 2010a, b; Ecker, 2017; Cauvet et al. 2019; Van't Westeinde et al. 2019; Walsh et al. 2021). For example, Bloss and Courchesne observed that girls with ASD exhibited additional sites of abnormalities compared to boys with ASD, notably in temporal regions (Bloss and Courchesnes 2007). In a sub-sample of ASD-discordant pairs, Van’t Westeinde et al. found extended increased thickness in the orbitofrontal regions, superior frontal gyrus, and intraparietal sulcus associated with restricted and repetitive behaviors and interests in females, whereas males only presented increased volume in the bilateral pallidum (Van't Westeinde et al. 2019). On a developmental point of view, females with ASD were found to display a more pronounced abnormal growth profile in more brain regions than males with ASD (Schumann et al. 2010a, b). In addition, one recent twin study on adolescents and young adults reported important brain correlates associated with autistic traits in females, and relatively few correlates in males (Cauvet et al. 2019). In women, higher autistic traits, also measured by the SRS, were associated with smaller volumes and surface areas in regions similar to those observed in the present study, notably the left superior temporal gyrus, the middle temporal gyrus, and the parahippocampus. Males had different patterns with fewer regions associated with autistic traits, including, however, the parahippocampus. Evidence for functional and microstructural studies also support more pronounced brain particularities associated with ASD (Walsh et al. 2021). Altogether these results suggest that females might possess protective mechanisms regarding ASD and require a higher detrimental load, including structural brain alterations, before developing clinically relevant levels of autistic traits. Effect of sex steroids on brain development may interact with ASD risk genes to produce these sex differences (Walsh et al. 2021; Ferri et al. 2018). Altogether, these results and hypotheses advocate for further studies focusing on brain correlates and trajectories associated with autistic traits and ASD in females.

The most important region highlighted in the present study is the left parahippocampus. Lower thickness of this region has been associated with ASD in the mega-analysis of van Rooij et al. (2018) but the authors did not report any effect of age. In addition, one previous study reported volumetric particularities in the parahippocampus associated with autistic traits, but the findings were of larger volume in the right parahippocampus (Focquaert and Vanneste 2015). The parahippocampal cortex has been associated with many cognitive processes, such as episodic memory, visuospatial processing, spatial representation and navigation, center-periphery of the visual field distinction, processing of emotional stimuli and treatment of auditory and odor stimuli (Aminoff et al. 2013). Aminoff et al. (2013) suggested that the parahippocampal cortex is part of a network of brain regions that processes contextual associations underlying these different cognitive processes. Interestingly, deficits in contextual information processing is present in ASD and a poor global processing, and consequently poor context processing, characterizes the Weak Central Coherence theory proposed to describe autistics behaviors (Ben-Yosef et al. 2017).

Also, in the present study, the communication sub-score was associated with slower volumetric decrease in the left superior temporal gyrus and the left pallidum. In ASD, abnormalities of these two regions have been largely reported (van Rooij et al. 2018; Shen et al. 2022; Sussman et al. 2015; Weerasekera et al. 2022). The superior temporal gyrus (STG) is involved in auditory processing, including language, but also in social cognition (Bigler et al. 2007). Part of the basal ganglia, the pallidum is involved in several important functions in the brain, including sensory control, motor programming, and reward-driven behaviors. In ASD, however, abnormalities of the pallidum have been more associated with restricted and repetitive behaviors and interests (Estes et al. 2011; Wilkes and Lewis 2018). A recent twin study reported increased volume of the bilateral pallidum associated with restricted and repetitive behaviors and interests in males but not in females (Van’t Westeinde et al. 2019).

In the present study, two regions were particularly associated with the social cognition dimension: the left middle temporal gyrus (MTG) and the right vmPFC/OFC. The PFC is the core brain region in the mentalizing network and is considered as a central hub of the “social brain” (Han et al. 2021). The vmPFC and OFC are involved in the emotional part of the theory of mind and are mainly responsible for understanding the emotional state of others (Abu-Akel and Shamay-Tsoory 2011). Larger GM volumes in these regions were found in individuals with a larger social network size (Kanai et al. 2012) and in those with higher mentalizing competence (Powell et al. 2012). Recently, a clinical trial observed that autistic children randomized in social cognitive skills groups demonstrated increased activity in the mPFC during an irony task that was associated with an improvement in social functioning on the SRS (Ibrahim et al. 2021). The left MTG is also involved in the social brain and the theory of mind. It is a critical node of the brain’s language network, and is notably involved in communicative-pragmatics, i.e., the ability to use language to convey meaning in a specific context by requiring inferential steps (Morese et al. 2022). More specifically, left MTG has been associated with comprehension of lexical ambiguity (Acheson and Hagoort 2013) and irony (Rapp et al. 2013). Several fMRI studies of healthy participants reported an activation of the left MTG for the correct understanding of irony (Bosco et al. 2017), semantic integration (Noppeney and Price 2004) or analysis of the coherence of a text (Morese et al. 2022). Left MTG could thus play a pivotal role in the understanding of complex communication. Finally, decreased connectivity between the orbitofrontal cortex and the MTG have been reported in adults with ASD (Cheng et al. 2017).

The strengths of this study are the relatively large and homogenous adolescent/young adult population, the use of confounding variable, such as IQ for the entire sample, the 8-year follow-up and the whole brain longitudinal protocol. The most important limitations regard the use of the SRS. First, the SRS has been shown to be relatively unspecific, with high score found in individuals with other mental or neurodevelopmental disorders (e.g.,Capriola-Hall et al. 2021; Reiersen et al. 2007). Second, in the present study, parent-reported SRS scores were measured at age 16, whereas imaging data were collected at age 14 and 22. However, autistic traits are known to be stable (Robinson et al. 2011a, b) even from childhood through early adulthood (Wagner et al. 2019).

## Conclusions

Overall, the present study demonstrated for the first-time specific brain trajectories, from mid-adolescence to young adulthood, associated with autistic traits in young women from the general population. These specific brain trajectories were observed in regions of the social brain and the reward circuit known to be involved in ASD. The present findings are in line with previous literature and could support the hypothesis of an earlier GM maturation associated with higher autistic traits in adolescence that normalizes in early adulthood. The absence of results in males support the hypothesis of previous studies that females might possess protective mechanisms regarding ASD and require a higher detrimental load, including structural brain alterations, before developing clinically relevant levels of autistic traits. Altogether, these results advocate for further studies focusing on brain correlates and trajectories associated with autistic traits and ASD in females.

## Data Availability

Imagen data can be requested here: https://imagen-project.org/the-imagen-dataset/.
